# Domain Adaptation of Synthetic Images for Wheat Head Detection

**DOI:** 10.3390/plants10122633

**Published:** 2021-11-30

**Authors:** Zane K. J. Hartley, Andrew P. French

**Affiliations:** 1 School of Computer Science, University of Nottingham, Nottingham NG8 1BB, UK; 2 School of Biosciences, University of Nottingham, Loughborough LE12 5RD, UK; andrew.p.french@nottingham.ac.uk

**Keywords:** plant phenotyping, computer vision, deep learning, domain adaptation

## Abstract

Wheat head detection is a core computer vision problem related to plant phenotyping that in recent years has seen increased interest as large-scale datasets have been made available for use in research. In deep learning problems with limited training data, synthetic data have been shown to improve performance by increasing the number of training examples available but have had limited effectiveness due to *domain shift*. To overcome this, many adversarial approaches such as Generative Adversarial Networks (GANs) have been proposed as a solution by better aligning the distribution of synthetic data to that of real images through domain augmentation. In this paper, we examine the impacts of performing wheat head detection on the global wheat head challenge dataset using synthetic data to supplement the original dataset. Through our experimentation, we demonstrate the challenges of performing domain augmentation where the target domain is large and diverse. We then present a novel approach to improving scores through using heatmap regression as a support network, and clustering to combat high variation of the target domain.

## 1. Introduction

Detection of individual wheat heads is a difficult challenge in computer vision. Counting or measuring plant organs is an important plant of the plant phenotyping pipeline and is crucial for yield estimation in an agricultural setting. In recent years, datasets of top-down and aerial photography of different crops have become common, and the specific challenges of these kind of images have become important problems in deep learning research. Wheat heads are especially difficult because of their small size and due to a high level of occlusion and blurring caused by their constant motion caused by wind.

Labelling datasets such as these presents a challenge since a given image may contain dozens of individual wheat heads that require annotation. By using synthetic data to supplement the existing dataset, we aim to improve scores while reducing the cost of manual annotation, as has been achieved in other phenotyping tasks [1].

Domain adaptation is the field of deep learning concerned with solving the domain gap problem. It is well established that neural networks trained on a given domain often perform poorly when applied to different domains even where they are visually similar. In problem spaces such as plant phenotyping, this kind of approach is highly relevant owing to the high diversity of different plants and plant varieties; even within the same species, it is common to see a domain shift between different datasets of the same species due to imaging location and setup, time of year, the age of the plant, etc. In the past 5 years especially, *synthetic to real* domain adaptation has become popular [2,3] since automatically generated data are cheaper than the collection of real images and do not need manual annotation. Increased popularization of adversarial approaches has contributed to this increase in popularity, with many GAN-based models being used for domain adaptation.

Further to this, the GWHC dataset reflects an enormous diversity of images from different institutions around the world, exhibiting different varieties of wheat at different stages of maturity. Datasets that feature these high levels of heterogeneity can prove more difficult for domain adaptation because of the greater range of distributions that must be aligned in order to allow for accurate predictions to be made across different test examples. Our method seeks to combat the effect of this by partitioning the training dataset prior to performing domain adaptation to enable us to produce multiple transformed images from each synthetic input image.

We combine this insight with an adversarial approach that is combined with a support network to guide the generator during training with an aim of ensuring that *synthetic to real* style transfer can still preserve wheat head geometry.

In summary, our main contributions are:

1. We present a novel approach to improving wheat head detection scores leveraging synthetic data with a domain adaptation architecture.
2. We demonstrate good performance on the GWHC using our methodology.
3. We release our synthetic wheat dataset, containing over 10,000 synthetic wheat images with bounding box annotations.

In Section 2, we present a literature review of object detection, current uses of synthetic data in plant phenotyping, as well as other approaches to the plant organ counting and the GWHC more generally. In Section 3, we describe our approach to using *synthetic to real* domain adaptation for wheat head detection as well as describing our experiments, results of which are then shown in Section 4. Finally, a full discussion of our work with regards to the results presented can be found in Section 5.

## 2. Related Work

### 2.1. Object Detection

Object detection is one of the most important computer vision tasks, aiming to predict locations for individual objects within an image. Early methods in this field use a variety of approaches, including different feature-based approaches [4,5], sliding window methods [6], and deformable parts-based models [7]. At present, there are two popular approaches to object detection seeing use. The *You Only Look Once* (YOLO) family of object detection networks [8,9] make their predictions in a single iteration per image, dividing an image into a grid and making predictions in each segment. Alternatively, architectures that use a two-stage approach to object detection use region proposal networks to identify regions of interest within an input image, from which a bounding box and classification label can be predicted. In our paper, we perform our object detection using a Region-based Convolutional Neural Network (R-CNN) model. We use the popular Detectron2 [10] implementation, which includes Mask R-CNN [11] object detection and segmentation architecture. We can therefore demonstrate the improvements bought about by our method using a standard detection approach.

### 2.2. Wheat Head Detection

Wheat head detection has received an increase in attention in recent years due to the release of the global wheat head detection dataset [12] and associated challenges; however, the detection and counting of individual ears of wheat has been of interest for some time. Originally (and still in many circumstances) wheat head counting was a manual task, performed by hand. Early interest in wheat head counting such as [13] uses a more traditional image processing pipeline to segment ears of wheat for counting; however, detection of individual plant organs has been limited to larger plant organs such as seen in [14], or entire crops such as [15], until very recently. The introduction of popular deep learning architectures for detection discussed above has allowed for recent work to focus on the domain specific challenges related to wheat head detection.

With the introduction of deep learning, more complex images of wheat heads could be analyzed more successfully. Khaki et al. [16] presented a lightweight model designed for mobile deployment that detects wheatheads using density and localization maps. Finally, at CVPPA 2021 Liu et al. [17] demonstrated their method using color transformation to reduce false negatives in wheat head detection.

### 2.3. Domain Adaptation

Attempting to solve the domain shift problem through domain adaptation has been proposed for a wide range of different problems in recent years. In computer vision, attempting to alter training images to more closely match a target domain is common, as seen in Zhang et al., where they present a method for using domain adaptation for the detection of different fruits using a CycleGAN [18] demonstrating transformation between different fruits for a detection task. Domain adaptation has proven popular across most of the main phenotyping tasks such as leaf counting via regression as shown in [19] and wheat head detection as shown in [3] and in our own work.

Domain adaptation is heavily related to transfer learning, and many papers use fine tuning to improve performance across domains. In Tapas et al. [20], fine-tuning is used to improve performance on the *Computer Vision Problems in Plant Phenotyping* (CVPPP) leaf segmentation challenge (https://www.plant-phenotyping.org/CVPPP2020, accessed on 28 August 2020). This is similar to a recent paper by Najafian et al., who presented a similar idea focusing on domain adaptation from simulated images for the GWHC [2], which combines a synthetic data approach with fine tuning using iterations of pseudo labelling new datasets.

In this paper, we present our methodology using similar domain adaptation concepts presented in the work above and apply a novel pipeline for training a detection network from synthetic data.

## 3. Materials and Methods

In this section, we describe our synthetic dataset and our pipeline for generating such images, our domain adaptation network, and the experiments we conducted using Detectron 2 as our task network.

### 3.1. Synthetic Data Creation

**Figure 1:**
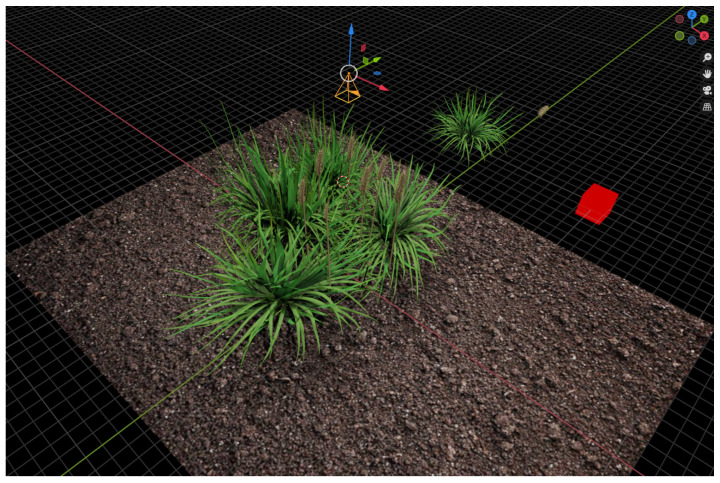
An image of our Blender setup, showing synthetic wheat heads dispersed among foliage and the camera used to capture each image positioned above the scene.

To perform domain adaptation, we created a pipeline to creating synthetic images that would allow us to supplement the existing GWHC dataset of real images used for our experiments. This pipeline uses Blender to randomly generate new 3D models of scenes containing a large number of wheat heads consisting of a generated stem and a hand crafted wheat ear that is added last to create the finished look of each plant. Each wheat stem was created using a custom Python extension that implements Lindernmayer Systems (L-systems), where natural-looking structures can be generated via expansion rules defined as a string [21]. For each image, we generate a randomly determined wind speed and direction, and the string used to generate the L-system for each stem in a given image will be modified based on these values. As such, while every crop will be slightly randomised, the overall scene is able to consistently model the strong bending due to wind force common in images of wheat fields. For each image, this process was repeated 30 to 60 times, before models of additional foliage were added to complete the background. We show an example of the scene in Blender in Figure 1, showing the camera positioned above a typical scene.

For each image, we were required to create accurate ground truth labels showing bounding boxes for each ear of wheat. In order to do this, we used Blender’s camera data combined with world coordinates for each wheat head to extract the values of the bounding box within the camera frame and exported these to a CSV file for each image. The final dataset $S_{I}$ contains over 5000 new images with over 100,000 new annotations, to be used in training for our experiments. Finally, to create additional labels for our heatmap support, we used OpenCV [22] to create Gaussian heatmaps for each image based on the extracted bounding box values creating a set of labels $S_{H}$; this process was repeated to create heatmaps for the real-world training images $R_{H}$ used as our target domain, which are supplied with bounding box labels.

### 3.2. CycleGAN with Heatmap Support

**Figure 2:**
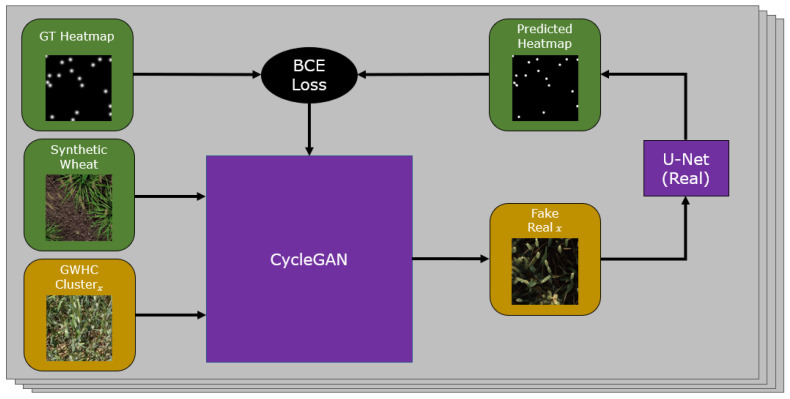
Our CycleGAN model with Gaussian heatmap support; this process is repeated for each cluster extracted from our target dataset (represented by the four layers in the figure), producing multiple outputs for each input synthetic image.

Our goal is to create new images that can be used for training our network by transforming our synthetic images to the *real* domain and thus solve the domain shift problem. We adapt a CycleGAN model shown in Figure 2 to predict Gaussian heatmaps of wheat head locations from the output of both the *real to synthetic* and *synthetic to real* generators. By doing this, we aim to preserve the locations and geometry of wheat ears in images transformed by the generators, as wheat heads added or removed will create inaccuracy in the predicted heatmaps. To do this, we extend the architecture with two lightweight UNets that predict heatmaps for the outputs of each generator. Our CycleGAN is set up with default parameters for training, and our synthetic dataset and the training split of the GWHC dataset are used as source and target domains respectively. All images are resized to 400 × 400 due to the high VRAM constraints of combining CycleGAN with additional models as support. Additionally, for all experiments we perform, all training images and testing images are resized to 400 × 400 to be consistent with GAN output images.

**Figure 3:**
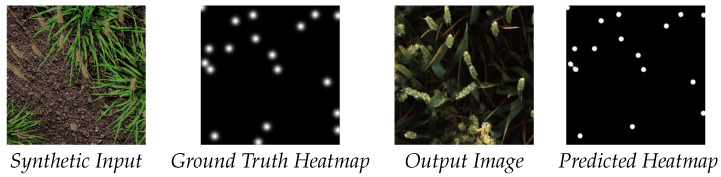
Examples of our heatmap-supported pipeline, showing input image and groundtruth paired with a corresponding output and predicted gaussian heatmap. High similarity between ground truth and predicted labels indicates wheat head location has been preserved during augmentation.

For each iteration, our model receives a source image $S_{I_{i}}$ and a target image $R_{I_{i}}$ and transforms each to the other’s domain, *synthetic* and *real*, respectively. The predicted heatmaps are compared against $S_{H_{i}}$ and $R_{H_{i}}$, and we apply a binary cross entropy loss to simultaneously train each UNet and enforce accurate translation of the wheat heads by the generator. An example of both the input and output images along with ground truth and the predicted heatmap can be seen in Figure 3. After training our model, we produce dataset H where all images from S have been converted by the *synthetic* and *real* generator. We also produce data C where we use an unmodified CycleGAN as a baseline to compare our extended model against.

### 3.3. Feature-Based Clustering to Improve Diversity of Generated Images

Using CycleGAN to convert images to the real domain using our *real* wheat head image dataset presents problems due to the heterogeneity of the images present in our target domain. Rather than producing images that represent the diverse range of images in the GWHC dataset, the generator instead learns a translation to an *average* wheat image representation. This is a problem we want to avoid as the average image is not a meaningful representation of real life.

**Figure 4:**
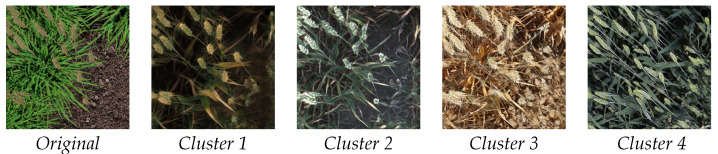
Examples of 4 images produced from a single synthetic image for our Heatmap-Supported 4 clusters experiment. Each cluster represents a different broad category of images from R; these subsets may be grouped by wheat species and growth stage, time of day, or other factors in how the image was captured.

To counter this, we apply a preprocessing step of splitting our dataset into distinct visual appearance clusters to be trained as separate targets for our generator. To do this, we use an pretrained InceptionV3 feature extractor to obtain a feature vector from each image in R. We then apply K-means clustering to group the images into clusters of similar images. We select a value of k = 4 to achieve the best compromise between maximising the number of clusters while ensuring enough images are present in each set to make CycleGAN viable. Increasing the value of k may lead to more diversity in the data produced; however, while we can easily change this value for every cluster, we must run additional models that are more computationally expensive and may lead to poorer results due to the smaller size of individual clusters. Visual inspection confirms that each cluster appears visually similar to other images in the same group. In our case, k = 4 makes sense due to the four main appearance modes in the dataset; examples of each modes can been seen in Figure 4.

For our experiments, we trained four of our extended CycleGAN models using each cluster as a target, respectively, testing this methodology both with and without heatmap support. Each model was then used to generate a *real* representation for each image in our synthetic dataset, in doing so quadrupling the amount of data available for training Detectron. By combining these four sets of augmented images, we create our final dataset K, which we use to obtain our final scores.

### 3.4. Experiments

(1) Real Only

Here, we establish a baseline performance achieved by using only the real data from our training split R of the GWHC dataset, containing over 3000 images. For all our tests, all images have been resized to 400 × 400 for consistency. We expect this set to perform well as it is already diverse and highly representative of the test split.

(2) Synthetic and Real

We evaluate the performance gain combining our synthetic dataset S with the real training data R but without GAN modification. Synthetic data have been leveraged to improve performance in a number of other domains; however, it is unlikely to have a significant impact on performance due to the significant domain shift between synthetic and real images.

(3) CycleGAN

Here, we evaluate the performance achieved using images from C, which have been augmented by an unmodifed CycleGAN, as our training set. We perform this experiment to verify that our support network improves scores against a suitable comparison. Due to cases where the cycleGAN either removes true wheat heads or incorrectly adds new ones (hallucinating heads where there should be none) to the image, we expect this network to perform poorly.

(4) Heatmap-Supported, No Real

We evaluate the performance when Detectron is trained using only images in H, generated by our CycleGAN with Gaussian regression support. We expect this network to perform well even without real images being included in the train set, demonstrating that our images are suitable replacements for real image for the purposes of training.

(5) Heatmap-Supported and Real

Here, we combine H with the *real* images from R. We expect this to boost performance on the network significantly compared with experiment 4 because of the inclusion of images from the target domain.

(6) Heatmap-Supported 4 Clusters, No Real

As described in Section 3.3, we create four subsets of the original training set to create converted datasets for each of the four targets using our extended model. We combine these four datasets into a combined set K. We expect this network to perform well, even when real images are not used to train detection due to images in K being a close likeness to images in our test set.

(7) Heatmap-Supported 4 Clusters and Real.

Finally, we combine K with R. We expect this model to perform the best of all the experiments listed as it combines all the advances of our approach plus real image data.

### 3.5. Training

Our CycleGAN with heatmap support models was run using standard CycleGAN settings. Both generators are extended with an additional model to predict Gaussian heatmaps of wheat head locations, both of which share their Adam optimisers and learning rates of $2\times 10^{-4}$ with their respective generator. These models were each trained for 100 epochs, after which GAN training performance began to degrade.

Our experiments were performed with an unmodified Detectron 2 using standard settings, trained on NVIDIA GTX Titan X (Pascal) GPUs for 60 epochs.

## 4. Results

In this section, we demonstrate the images produced by our domain adaptation models as well as presenting the results of the experiments described above. We compare our results against a baseline achieved using only real image in our training dataset.

### 4.1. Domain Adaption Results

**Figure 5:**
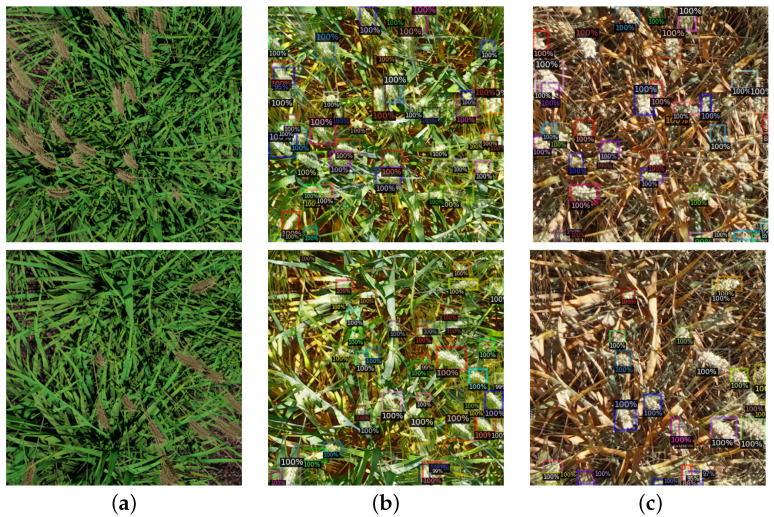
Comparison of synthetic images (**a**) augmented with unmodified CycleGAN model; (**b**) and CycleGAN with heatmap support (**c**). Images in column C demonstrate a realistic colour and higher contrast between wheat heads and background. We observe that for the image from column B (dataset C) more predictions are made, leading to a lower accuracy. (**a**) *Synthetic*; (**b**) *CycleGAN*; and (**c**) *Heatmap Supported CycleGAN*.

In Figure 5, we compare augmentation between a conventional CycleGAN and our extended model. We see that visually the images produced by both models look very different. The conventional CycleGAN image is more saturated than images produced by the supported network, though it is likely more imperceivable changes are being made by the generator that are difficult to identify. We also observe that Detectron makes more predictions for the image produced by the unchanged CycleGAN model, and, as shown in Table 1, this leads to worse overall performance. It is difficult to infer exactly why this is due to the black-box nature of deep learning models. Overall, there was a small positive performance increase thanks to our support network component, but the impact was less important to our overall result than we originally expected. This may be due to the unmodified CycleGAN proving more effective at maintaining geometry than we had initially hypothesised.

Figure 4 shows an output example of our CycleGANs used to create dataset K for experiments 6 & 7 along with an input image. Here, we can see examples of how a single synthetic image from our source domain can be transformed to a number of different domains each matching a subset of the overall *real* target domain. In all cases, wheat heads appearing in the synthetic image, and by extension their annotations, have had locations maintained well and appear realistic while maintaining their geometry. Some additional wheat heads are observed to be added especially in cluster 4, but this is less so than we would expect to see using a conventional CycleGAN.

### 4.2. Wheat Detection

For each of our experiments, we evaluate our results using both Mean IoU of bounding boxes and Mean Euclidean Distance between center points of the boxes. We report results of all our experiments in Table 1. In experiment 1, the baseline achieved by training Detectron with only R performed well as expected. Experiment 2 exceeded our expectations by increasing the baseline score, suggesting the synthetic data we have created did a good job at imitating the target domain and that this had a less significant domain shift problem than would be expected from most synthetic datasets.

In experiments 3, 4, and 6, we compare the performances achieved by C, H, and K before introducing images from R. We observe that the heatmap-supported network offers a small improvement over an unmodified CycleGAN and that our clustering approach leads to a much more significant improvement.

Finally, in experiments 5 & 7 we compare scores achieved when we add the real images from R to H and K, respectively. Surprisingly, experiment 5 shows only a small improvement, not even beating our results from experiment 2 using unaltered synthetic data. Experiment 7 achieved our highest scores in most metrics, beating our baseline by nearly 5%.

**Figure 6:**
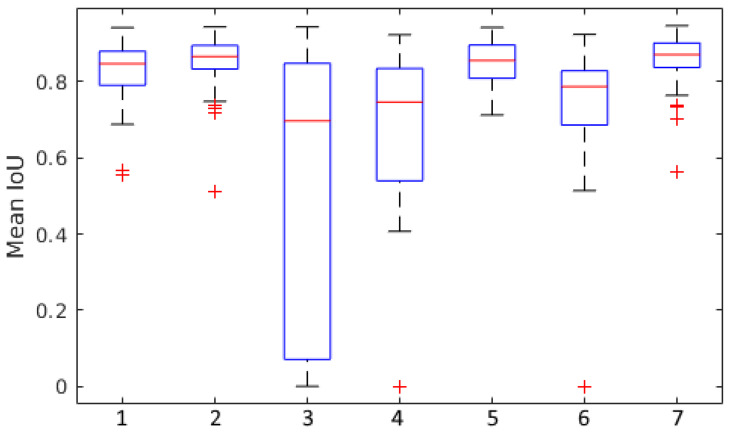
A boxplot visualisation of data for each of our Mean IoU scores. Numbers along the x-axis correspond to experiment numbers described in Section 3.4.

In Figure 6, we show further data visualising the results of our experiments. Here, we see that each experiment without real images during training has significantly higher numbers of outliers. Some of the images included in our test set have very small numbers of wheat heads, and poor performance on these images may have significantly increased the spread of the data in these cases. We also observe that experiments 2, 5, and 7 (which include real data during training) all have very low standard deviations shown in Table 1, in addition to their very high scores overall. This suggests consistently high performance of the proposed approach across all test instances.

## 5. Discussion

### 5.1. Analysis of Results

In our results, we see that the difference between experiments 2 and 5 is extremely small, with experiment 2 (using unmodified synthetic images) having better scores in both Mean IoU and Euclidean Distance. We hypothesise that with heatmap support alone, there is still some loss of accuracy, which we expect might be caused by domain adaptation failures. It is, however, likely that the strong performance of S in experiment 2 indicates that we have succeeded in producing a high-quality synthetic wheat dataset well suited to use as a foundation for further enhancements.

We observe that in experiments 6 and 7, the addition of our clustering approach produces the best scores in both metrics. Experiment 6 is noteworthy for having the best score achieved for any method that did not use real images at all, suggesting that an unsupervised adaptation of our methodology might be viable. Similarly, experiment 7 achieving improvements over all other methods shows the efficacy of our overall approach.

A further take-home message is just how beneficial adding real images is, versus synthetic images alone (experiment 4 vs. 5; experiment 6 vs. 7). Performance improvements of at least 0.2 can be found by combining real images into the set. This highlights the value of any labelled real images that can be added to the train set.

As discussed in Section 3.2, for all the experiments presented in Table 1 we used a confidence threshold of 0.7. During additional experimentation, we observed that for experiments 4 and 6, where no real images were used, higher scores could be increased by lowering the confidence threshold to 0.4, although this would produce much lower scores for all experiments with real images from R. As images from these networks were never exposed to any real images during training, it is likely that it has lower confidence of any predictions made on our test set, and as such the high 0.7 threshold causes some correct predictions to be discarded. However, it is clear that optimising this per experiment, although feasible for a particular real-world problem, would not be considered fair, so was kept constant for the results in Table 1.

### 5.2. Future Work

The method presented in this paper is well suited to being applied to other detection tasks, especially where data availability is a limitation. Especially in fields like plant phenotyping, where data for different crop varieties is often unavailable, we believe our method could be applied to detection of disease foci, as well as detection and counting of plant organs, leaves, or fruit.

## 6. Conclusions

In this paper, we have presented a new approach to improving scores on wheat head detection using a supported CycleGAN and a novel clustering method that allows us to increase the quantity of our training data while also increasing diversity in our data produced by domain adaptation. Our results show our methodology improves scores when tested on unseen images from the GWHC dataset, compared to a baseline score set by real images from the same dataset. Our method is also highly generalizable and could be easily adapted to work on other plant phenotyping problems, especially where smaller quantities of training data are available.

## Figures and Tables

**Table 1:** Results of the experiments described in Section 3.4, showing results for Mean IoU (higher is better) and Mean Euclidian Distance (lower is better) reported for test split of 100 GWHC images. The best scores in both metric were achieved by experiment 7, which has been highlighted.

Experiment	Mean IoU ± SD	Mean Euclidean Distance
(1) Real Only	0.8262 ± 0.07	13.9079
(2) Synthetic and Real	0.8568 ± 0.06	10.7702
(3) CycleGAN, No Real	0.5625 ± 0.35	16.9433
(4) HM Support, No Real	0.5987 ± 0.33	12.7729
(5) HM Support and Real	0.8497 ± 0.06	11.1276
(6) HM Support 4 Clusters, No Real	0.6622 ± 0.30	14.7164
(7) HM Support 4 Clusters and Real	**0.8642** ± 0.06	**10.5617**

## Data Availability

The data presented in this study are openly available at plantimages.nottingham.ac.uk.

## References

[1] Fei Z., Olenskyj A.G., Bailey B.N., Earles M. Enlisting 3D Crop Models and GANs for More Data Efficient and Generalizable Fruit Detection. Proceedings of the IEEE/CVF International Conference on Computer Vision.

[2] Najafian K., Ghanbari A., Stavness I., Jin L., Shirdel G.H., Maleki F. A Semi-Self-Supervised Learning Approach for Wheat Head Detection Using Extremely Small Number of Labeled Samples. Proceedings of the IEEE/CVF International Conference on Computer Vision.

[3] Ayalew T.W., Ubbens J.R., Stavness I. (2020). Unsupervised domain adaptation for plant organ counting. European Conference on Computer Vision.

[4] Dalal N., Triggs B. Histograms of oriented gradients for human detection. Proceedings of the 2005 IEEE Computer Society Conference on Computer Vision and Pattern Recognition (CVPR’05).

[5] Lienhart R., Maydt J. An extended set of haar-like features for rapid object detection. Proceedings of the IEEE International Conference on Image Processing.

[6] Ferrari V., Fevrier L., Jurie F., Schmid C. (2008). Groups of Adjacent Contour Segments for Object Detection. IEEE Trans. Pattern Anal. Mach. Intell..

[7] Felzenszwalb P.F., Girshick R.B., McAllester D., Ramanan D. (2010). Object Detection with Discriminatively Trained Part-Based Models. IEEE Trans. Pattern Anal. Mach. Intell..

[8] Redmon J., Farhadi A. (2018). Yolov3: An incremental improvement. arXiv.

[9] Bochkovskiy A., Wang C.Y., Liao H.Y.M. (2020). Yolov4: Optimal speed and accuracy of object detection. arXiv.

[10] Wu Y., Kirillov A., Massa F., Lo W.Y., Girshick R. (2019). Detectron2. https://github.com/facebookresearch/detectron2.

[11] He K., Gkioxari G., Dollár P., Girshick R. Mask r-cnn. Proceedings of the IEEE International Conference on Computer Vision.

[12] David E., Madec S., Sadeghi-Tehran P., Aasen H., Zheng B., Liu S., Kirchgessner N., Ishikawa G., Nagasawa K., Badhon M.A. (2020). Global Wheat Head Detection (GWHD) dataset: A large and diverse dataset of high-resolution RGB-labelled images to develop and benchmark wheat head detection methods. Plant Phenomics.

[13] Cointault F., Gouton P. Texture or color analysis in agronomic images for wheat ear counting. Proceedings of the 2007 Third International IEEE Conference on Signal-Image Technologies and Internet-Based System.

[14] Pape J.M., Klukas C. (2014). 3-D histogram-based segmentation and leaf detection for rosette plants. European Conference on Computer Vision.

[15] Weiss U., Biber P. (2011). Plant detection and mapping for agricultural robots using a 3D LIDAR sensor. Robot. Auton. Syst..

[16] Khaki S., Safaei N., Pham H., Wang L. (2021). Wheatnet: A lightweight convolutional neural network for high-throughput image-based wheat head detection and counting. arXiv.

[17] Liu C., Wang K., Lu H., Cao Z. Dynamic Color Transform for Wheat Head Detection. Proceedings of the IEEE/CVF International Conference on Computer Vision.

[18] Zhang W., Chen K., Wang J., Shi Y., Guo W. (2021). Easy domain adaptation method for filling the species gap in deep learning-based fruit detection. Hortic. Res..

[19] Giuffrida M.V., Dobrescu A., Doerner P., Tsaftaris S.A. Leaf counting without annotations using adversarial unsupervised domain adaptation. Proceedings of the 2019 IEEE/CVF Conference on Computer Vision and Pattern Recognition Workshops (CVPRW).

[20] Tapas A. (2016). Transfer learning for image classification and plant phenotyping. Int. J. Adv. Res. Comput. Eng. Technol. (IJARCET).

[21] Prusinkiewicz P. Graphical applications of L-systems. Proceedings of the Graphics Interface.

[22] Itseez (2015). Open Source Computer Vision Library. https://github.com/itseez/opencv.

